# Larval trematode communities in *Radix auricularia *and *Lymnaea stagnalis *in a reservoir system of the Ruhr River

**DOI:** 10.1186/1756-3305-3-56

**Published:** 2010-06-24

**Authors:** Miroslava Soldánová, Christian Selbach, Bernd Sures, Aneta Kostadinova, Ana Pérez-del-Olmo

**Affiliations:** 1 Institute of Parasitology, Biology Centre v.v.i., Academy of Sciences of the Czech Republic, Branišovská 31, 370 05 České Budějovice, Czech Republic; 2 Department of Applied Zoology/Hydrobiology, University of Duisburg-Essen, Universitätsstrasse 5, D-45141 Essen, Germany

## Abstract

**Background:**

Analysis of the data available from traditional faunistic approaches to mollusc-trematode systems covering large spatial and/or temporal scales in Europe convinced us that a parasite community approach in well-defined aquatic ecosystems is essential for the substantial advancement of our understanding of the parasite response to anthropogenic pressures in urbanised areas which are typical on a European scale. Here we describe communities of larval trematodes in two lymnaeid species, *Radix auricularia *and *Lymnaea stagnalis *in four man-made interconnected reservoirs of the Ruhr River (Germany) focusing on among- and within-reservoir variations in parasite prevalence and component community composition and structure.

**Results:**

The mature reservoir system on the Ruhr River provides an excellent environment for the development of species-rich and abundant trematode communities in *Radix auricularia *(12 species) and *Lymnaea stagnalis *(6 species). The lake-adapted *R. auricularia *dominated numerically over *L. stagnalis *and played a major role in the trematode transmission in the reservoir system. Both host-parasite systems were dominated by bird parasites (13 out of 15 species) characteristic for eutrophic water bodies. In addition to snail size, two environmental variables, the oxygen content and pH of the water, were identified as important determinants of the probability of infection. Between-reservoir comparisons indicated an advanced eutrophication at Baldeneysee and Hengsteysee and the small-scale within-reservoir variations of component communities provided evidence that larval trematodes may have reflected spatial bird aggregations (infection 'hot spots'). Two life history groupings of dominant species, the 'cyprinid' and 'anatid' parasites, that depict two aspects of progressive eutrophication in this mature reservoir system, were identified.

**Conclusions:**

We conclude that trematode communities in the lake-adapted *R. auricularia *are better suited for monitoring the effect of environmental change on host-parasite associations in the reservoir system on the Ruhr River and other similar systems due to the important role of this host in trematode transmission in lakes. Whereas variations in trematode community diversity and abundance may indicate the degree of eutrophication on a larger scale (among reservoirs), the infection rates of the two life history groups of dominant species, the 'cyprinid' and 'anatid' assemblages, may be particularly useful in depicting environmental variability, eutrophication effects and infection 'hot spots' on smaller spatial scales.

## Background

Snail-trematode systems have long been the focus of medical, evolutionary and ecological research, the latter resulting in a large body of literature on factors structuring trematode communities (reviewed in [1,2]). A community of sometimes phylogenetically distant larval trematodes in a snail host provides indication of co-evolved host-parasite compatibility and trophic links at different levels within an ecosystem. Since trematode communities in snails reflect the richness and abundance of free-living assemblages [3] they may appear good indicators of ecosystem health and anthropogenic pressure (see e.g. [4] and references therein). However, the community line of research depends on taxonomic expertise and the availability of information on trematode life-cycles which are highly diverse, especially in freshwater systems.

Research on trematode life-cycles originated in Germany with the remarkable works of Nitzsch [5], La Valette [6] and Lühe [7] and has flourished in the last century yielding knowledge on more than 177 species whose freshwater life-cycles can be completed in the country (see references in Faltýnková & Haas [8]). In spite of the vast amount of life-cycle data, only two surveys have been conducted since the 1970's dealing with trematode distribution in Germany. Loy & Haas [9] observed no change in the prevalence of larval parasites in *Lymnaea stagnalis *(L.) over 20 years and Faltýnková & Haas [8] revealed a diverse larval trematode fauna in molluscs (31 species) and the same spectrum of the "typical" for Central Europe species as that observed some 100-150 years ago by La Valette [6] and Lühe [7]. Both studies were predominantly carried out on old fishponds existing in the area for centuries and included the ponds of the Aischgrund lowland near Erlangen encompassing a nearly 200 ha natural reserve area hosting an exceptionally rich and abundant avian fauna (more than 250 species, [10]). Further, both studies report data from pooled either long-term (over 20 years) sampling of a single mollusc species [9] or short-term opportunistic collection of a wide array of hosts in a range of habitats in the south of Germany (Bavaria) [8]. The high diversity and stability of the trematode fauna in molluscs revealed by these authors may therefore, reflect the maturity and stability of the ecosystems studied in the south of Germany or the nature of the traditional faunistic approach to summarising data over time and space. However, studies at the community level associated with well-defined aquatic ecosystems in Germany that would help test these suggestions are still lacking. A parasite community approach would not only substantially advance our understanding of the parasite diversity in undisturbed freshwater habitats but would be essential for the assessment of parasite response to anthropogenic pressures in urbanised areas which are typical on a European scale.

The creation of large freshwater impoundments is one of the most dramatic anthropogenic impacts on both the natural environment and wildlife parasite fauna [11]. The responses of parasites to reservoir formation are not uniform both with respect to higher taxonomic groupings and/or life-cycle strategies utilised, and the age of reservoir (see Morley [11] for an extensive review). However, the majority of published data suggest that, in the long-run, the creation of dam reservoirs results in human-induced facilitation of trematode life-cycles. Thus, water resources development projects have led to increase in the prevalence and outbreaks of human schistosomiasis worldwide and are considered an important risk factor for human health (reviewed in [12]) and large dam constructions in the former Soviet Union have caused severe problems due to increased transmission of important fish pathogens of the genus *Diplostomum *[13]. However, analyses of how environmental change in mature reservoirs and mollusc-parasite systems are related, are still lacking.

The Ruhr catchment area in western Germany is emblematic, with the industrial pollution in the past and the vast anthropogenic impact exerted by the dense population associated with a large number of waste-water treatment plants in the catchment area. In this area the Ruhr River Association (Ruhrverband) has been running a complex water reservoir system for nearly a century which comprises a number of water structures, mostly dams and weirs, built on the Ruhr River and its tributaries which regulate the water system. A reservoir system affects the river flow making backwater influences inevitable and induces variability in hydrological conditions causing alterations in aquatic communities which, in turn, may affect levels of parasitism in potential available hosts.

This paper is the first to apply a community approach to the diversity of larval parasites in two species of lymnaeid snail, *Radix auricularia *(L.) and *L. stagnalis *in Germany and, to the best of our knowledge, in aquatic habitats of Central Europe. It is based on sampling in four mature (30-80 year-old) interconnected reservoirs of the Ruhr River, North Rhine-Westphalia, where these snails appear to be the predominant element of the molluscan fauna. We characterise the larval trematode fauna at the reservoir level and focus on small-scale among and within-reservoir variations in parasite prevalence and component community composition and structure, using distinct representative samples of both hosts.

## Methods

### Study area

**Figure 1:**
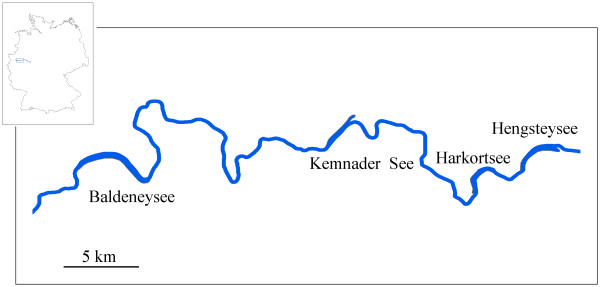
**Map of the area and the reservoir system studied on Ruhr River**.

Molluscs were sampled at four out of the five artificial reservoirs on the river Ruhr: Hengsteysee, Harkortsee, Kemnader See and Baldeneysee (see map in Fig. 1 and Table 1 for details). Baldeneysee is the largest of the four, noticeably longer and with more than double the volume of the other reservoirs which are of similar size (Table 1). All reservoirs were constructed (three as early as the 1930's) as deposit reservoirs of sediments from the inflowing rivers Lenne and Volme and natural water purification. With the construction and development of more effective sewage treatment plants along the Ruhr the initial function of water purification has become secondary; currently the reservoirs of the Ruhr River serve for drinking water and hydropower supply and recreation. The shallow nature of these water bodies has gradually resulted in volume loss due to the deposition and accumulation of sediments. As a restoration measure, sediment removal has been carried out in all reservoirs except Kemnader See during the last 20 years [14].

**Table 1:** General characteristics of the study reservoirs, water quality data recorded during sampling and sample sizes for *R. auricularia *and *L. stagnalis* (ranges followed by means in parentheses).

		Baldeneysee	Hengsteysee	Harkortsee	Kemnader See
**Reservoir data**	Construction (year)	1931 - 1933	1927-1929	1931	1979
	Length (km)	7.8	4.2	3.2	3.0
	Width (m)	355	300	335	420
	Surface area (ha)	264	136	137	125
	Mean depth (m)	3.1	2.0	2.2	2.4
	Mass development of E. *nuttallii*	-	+	+	+

**Water quality data**	Dissolved oxygen concentration (mg/L)	4.45-11.07 (8.09)	9.1-16.35 (11.07)	8.08-11.02 (9.56)	7.24-8.36 (7.87)
	pH	7.1-7.8	7.3-8.7	7.5-7.8	7.3-7.6
		(7.4)	(7.8)	(7.6)	(7.5)
	Conductivity (μS/cm)	390-615	341-478	438-483	415-658
		(505)	(406)	(461)	(527)
	Temperature (^°^C)	9.8-18.2	9.3-18.2	13-18.6	9.9-15.6
		(15.0)	(14.2)	(15.8)	(13.9)

**Snail data**	***Radix auricularia***				
	Total no. of snails (pooled samples)	169	195	22	44
	Mean shell height (mm)	15.6 ± 3.5	19.1 ± 3.5	11.9 ± 4.9	17.2 ± 5.1
	No. of distinct samples used (no. of snails)	4 (n = 137)^a^	4 (n = 166)^b^	1 (n = 20)^c^	
	***Lymnaea stagnalis***				
	Total no. of snails (pooled samples)	135	11	23	16
	Mean shell height (mm)	36.3 ± 8.9	31.3 ± 8.9	38.9 ± 8.7	34.3 ± 14.4
	No. of distinct samples used (no. of snails)	3 (n = 110)^d^		1 (n = 22)^e^	

**Bird data**	*Fulica atra*	> 60/80-100	> 100	> 100	> 50
	*Anas platyrhynchos*	15/20/50	+	+	+
	*Cygnus olor*	2/6/30	40	2	3
	*Phalacrocorax carbo*	6	5	+	
	*Larus ridibundus*	10	+	+	> 40
	*Branta canadensis*			+	20
	*Alopochen aegyptiacus*				2
	*Podiceps cristatus*			+	2
	*Tachybaptus ruficollis*	1			
	*Ardea cinerea*				1

Bird observations (counts where possible) were carried out on 20.11.2009 (multiple numbers represent counts at different sampling sites within a reservoir; see maps and photos in Additional file 1)

^a ^Sampling sites coded B1, B3a, B3b and B6;^b^Sites coded He1, He2, He3a and He6a;^c^Site Ha1;^d^Sites B1, B3a and B4;^e^Site Ha1 (see maps in Additional file 1);+ present but not counted.

Six run-of-the-river hydroelectric plants operate in the section of the Ruhr between the four reservoirs and one pumped-storage hydropower plant is operating at Hengsteysee. Power plant operation results in regular variations in the reservoirs' surface elevation associated with pumping and generation (e.g. of 0.5-0.7 m in Hengsteysee which serves as the lower reservoir of the pumped-storage hydropower plant, [14]); the inflow of warmer water into the post-dam reservoirs is another factor that may introduce changes of aquatic communities.

One setback associated with the shallowness of the reservoirs is the excessive growth of aquatic plants, a problem existing since the 1930's. Of particular recent concern is the mass development of the invasive macrophyte *Elodea nuttallii *(Planch.), native to North America which is the most widespread alien aquatic plant species in North Rhine-Westphalia causing problems in many lakes by covering almost the total water body and forming dense mats up to the water surface. Since its first mass occurrence in Harkotsee in 1994, massive infestations occurred in this reservoir in summer of 2000 and spread in the adjacent Hengsteysee and Kemnader See in the following years so that large surface areas were covered by macrophytes in summer (e.g. > 50% of the surface area, Hengsteysee; > 45%, Harkortsee; > 30%, Kemnader See, observed in aerial surveys in 2005; after a recession in 2006 and 2007 large surface areas of the reservoirs were infested again in 2008, see [14]). Although local patches of *E. nuttallii *were first observed in 2006, mass development of this waterweed has not yet occurred in Baldeneysee to the same extend as in the other reservoirs perhaps due to the higher turbidity caused by phytoplankton in this reservoir. However, large scale infestations were observed in this reservoir in 2008, with more than 13% of its surface covered by *E. nuttallii *[15].

The study area provides suitable habitats for a range of waterfowl, with many birds overwintering on the reservoirs. The spread of *E. nuttallii *may have contributed to providing abundant food source for plant-grazing birds such as swans, coots and mallards which have colonized the reservoirs [14]. Moreover the bird sanctuary Heisingen at Baldeneysee (*Vogelschutzgebiet Heisinger Bogen*) offers a protective environment for both fish-eating (e.g. *Phalacrocorax carbo*, *Larus ridibundus*, *Podiceps cristatus, Tachybaptus ruficollis, Ardea cinerea, Alcedo atthis*) and plant-grazing birds (e.g. *Cygnus olor, Fulica atra, Anas platyrhynchos, Branta canadensis, Alopochen aegyptiacus*) amongst others. Although no planned bird counts were carried out during our study, all these species were registered and documented in a visit to the sampling sites (see Table 1 for semi-quantitative data and Additional file 1 for detailed maps of the sampling sites and the areas of bird aggregations).

### Data collection

A total of 430 *R. auricularia *and 185 *L. stagnalis *was collected comprising 23 distinct snail samples (16 and 7, respectively) at different sites of the four reservoirs (labelled in the maps in Additional file 1). Snails were collected with hand-nets in the aquatic vegetation or picked by hand on stones along the shore of the reservoirs during 7 sampling trips in September-October 2009. The main bulk of samples was collected on 23 September (5 samples of *R. auricularia *and 2 of *L. stagnalis*) and 26 September (3 samples *R*. *auricularia *and 4 of *L. stagnalis*). Two samples of *R. auricularia *were collected on 5, 8 and 22 October; one sample on 12 and 19 October; and one sample of *L. stagnalis *was collected on 8 October.

Although sampling was not quantitative, the sample sizes (range 8-76) reflected the abundance of both hosts at the sampling sites. To reduce the bias due to small sample size we used only samples comprising more than 20 snails for quantitative comparisons (hereafter referred to as reduced dataset, a total of 13 samples comprising 323 *R. auricularia *and 132 *L. stagnalis*, thus representing 75% and 71% of the total samples, respectively, see breakdown by reservoir in Table 1). Snails were measured, labelled, placed in individual containers with dechlorinated tap water under a light source and screened for patent infections (*i.e*. cercarial emission) within 48 h after their transfer to the laboratory. Cercariae were identified alive using the keys of Faltýnková et al. [16,17] and other relevant primary sources [18,19]. Samples of selected species were fixed in molecular grade ethanol for DNA isolation and sequencing.

During each sampling trip the water temperature (°C), and three water quality parameters were measured at each site: dissolved oxygen concentration (mg/L) measured with Oxi 1970i, Wissenschaftlich-Technische Werkstätten GmbH, Weilheim, Germany (WTW); pH (measured with pH 320, WTW) and conductivity (°S/cm) (measured with Cond 315i; WTW). All measurements were taken at a depth of approximately 0.5-0.7 m at 1-2 m distance from the shore (ranges for multiple sites at each reservoir provided in Table 1).

### Data analysis

The effect on the probability of infection of snail size, reservoir of origin and the natural environmental variables measured at each site was assessed by a backward stepwise logistic regression (generalized linear models custom design with binomial distribution and logit link function in Statistica v.6, StatSoft, Inc., Tulsa, OK, USA) for both snail hosts. The dependent binomial variable "infection" was coded 0 (uninfected) and 1 (infected); one categorical ("reservoir" coded 1-4) and five continuous ("snail size","dissolved oxygen concentration", "temperature", "conductivity", and "pH") variables were used as covariates. Cases with missing data (e.g. 21 "large" infected *R. auricularia *of the first sampling died and were accidentally discarded without measuring) were excluded from regression analysis. This resulted in the lack of infected snails in the data for Harkortsee; regression for *R. auricularia *was, therefore, carried out for three of the reservoirs.

Species with prevalence higher than 10.0% in at least one community of the reduced dataset (*i.e*. sample size > 20) were considered dominant. Variations in levels of infection (the overall prevalence of larval trematode infection and the prevalences of the dominant species) of both snail hosts at the reservoir level were examined by contrasting pooled data across all of the sampling sites within a reservoir. At the component community level, only the data from distinct samples (n > 20 snails) within reservoirs were used. Assessment of intra-reservoir variability of larval communities was thus restricted to Baldeneysee (both hosts) and Hengsteysee (*R. auricularia *only; see Table 1 for details). Parasite prevalences were compared using Fisher's exact test in Quantitative Parasitology 3.0 [20]. Non-parametric tests (Mann-Whitney test, Spearman correlataions) were carried out with Statistica v.6. Component community composition analyses [non-metric multi-dimensional scaling (MDS) ordination and randomization tests on similarity matrices (ANOSIM); both based on Bray-Curtis index values] were carried out with PRIMER v6 software [21].

## Results

### Snail populations and natural environmental variables

Snail populations of *R. auricularia *were markedly more abundant at Baldeneysee and Hengsteysee whereas those of *L. stagnalis *had higher density at the former only (Table 1). Mean snail size ranged from 11.9 to 19.1 mm for *R. auricularia *and between 31.3 mm and 38.9 mm for *L. stagnalis*. The sample size for the latter species only was strongly correlated with the water temperature at the time of sampling (r_s _= 0.772, p < 0.05) whereas the size of the samples of *R. auricularia *exhibited a significant association with water pH (r_s _= 0.456, p <0.05). These correlations did not hold when tests were carried out on the reduced dataset of distinct samples considered for further quantitative comparisons.

Overall, natural environmental parameters varied within overlapping ranges among reservoirs (Table 1). Water temperature ranged between 9.3°C and 18.6°C depending on the day and time of collection. The hydrogen ion concentration was within the range providing protection for the aquatic life (6.0 to 9.0). The concentration of dissolved oxygen showed generally high (> 7.0 mg/L) levels and exhibited a strong negative association with water conductivity (r_s _= -0.665, p < 0.05). There were significant differences between reservoirs with respect to oxygen concentration only (Kruskal-Wallis H_(3,19) _= 8.80, p = 0.032) with highest levels observed at Hengsteysee (Table 1). Conductivity varied within the range normally found within the Ruhr catchment area.

### Larval communities in *Radix auricularia*

A total of 20.9% of all collected *R. auricularia *had patent larval trematode infections. Snail populations were very abundant in Hengsteysee and Baldeneysee where the overall prevalence of infection was notably high (28.2 and 18.3%, respectively, Table 2). This contrasted with the very low population density and overall prevalence recorded at Harkortsee and Kemnader See (4.6%). Although sample sizes varied between sites (20-76 snails) no significant correlation with the prevalence of infection was detected.

**Table 2:** Prevalence of the larval trematodes (% infected snails in the pooled samples) in the two hosts studied in the four Ruhr reservoirs and significance of differences (p) for between-reservoir comparisons (contrasts in overall prevalence and the prevalence of the dominant species).

		*Radix auricularia*					*Lymnaea stagnalis*				
**Trematode species**	**Final hosts**	**Baldeneysee**	**Hengsteysee**	**Harkortsee**	**Kemnader See**	**p**	**Baldeneysee**	**Hengsteysee**	**Harkortsee**	**Kemnader See**	**p**

Diplostomum spathaceum*	GUL	0.59	5.64 (1)	-	4.55	0.027	-	-	-	-	-
Trichobilharzia franki*	ANA	0.59	8.72 (2)	-	-	0.0001	-	-	-	-	-
Paryphostomum radiatum*	COR	3.55 (1)	4.10 (1)	-	-	ns	-	-	-	-	-
Echinoparyphium recurvatum*	ANA	11.24 (3)	4.10 (1)	-	-	0.007	1.48	-	-	-	-
Opisthioglyphe ranae*	AM	1.18 (1)	-	-	2.27	ns	-	-	-	6.25	-
*Plagiorchis elegans*	VAR	-	0.51	4.55	-	-	0.74	-	-	-	-
*Australapatemon burti*	ANA	-	1.03	-	-	-	-	-	-	-	-
*Echinostoma*sp.	ANA	0.59	1.54	-	-	-	-	-	-	-	-
*Hypoderaeum conoideum*	ANA	-	1.03	-	-	-	-	-	-	-	-
*Isthmiophora melis*	MAM	-	1.03	-	-	-	-	-	-	-	-
*Notocotylus attenuatus*	ANA	0.59	0.51	-	-	-	-	-	-	-	-
*Tylodelphys clavata*	GRE	-	1.03	-	-	-	-	-	-	-	-
Diplostomum pseudospathaceum*	GUL	-	-	-	-	-	3.70	9.10	17.39 (1)	12.50	0.036
*Echinostoma revolutum*	ANA	-	-	-	-	-	5.19	-	4.35	-	-
*Trichobilharzia szidati*	ANA	-	-	-	-	-	2.96	-	-	-	-

Overall prevalence (%)		18.3	28.2^a^	4.6	4.6^b^	0.0001	14.1	9.1	21.7	18.8	ns
No. of species in component communities (range)		1-4	1-7	1	-	-	1-2	-	2	-	-
No. of dominant species in component communities (range)		0-2	0-3	-	-	-	-	-	1	-	-

Dominant species are indicated by an asterisk and the number of communities where dominant given in parentheses following the overall prevalence. Abbreviations for the definitive host groups of trematodes: AM, amphibians; ANA, anatids; COR, cormorants; GRE, grebes; GUL, gulls; MAM, small mammals; VAR, various lariform and passeriform birds.

^a^two double infections; ^b^one double  infection.

*R. auricularia *in the Ruhr River reservoirs was infected with 12 species of trematodes, nearly half belonging to the family Echinostomatidae (five species including a new species of *Echinostoma *of the "*revolutum*" group detected by molecular methods, Kostadinova, unpublished data). The other seven species represent six families: Diplostomidae (2 species) and Notocotylidae, Plagiorchiidae, Schistosomatidae, Strigeidae and Telochiidae (1 species each; see Table 2). Three double infections were found: *Diplostomum spathaceum *- *Echinostoma *sp. (one snail from Hengsteysee, sample He2); *D. spathaceum *- *Paryphostomum radiatum *(one snail from Hengsteysee, sample He3b) and *D. spathaceum *- *Opisthioglyphe ranae *(one snail from Kemnader See, sample K1a). Six of the trematodes parasitising *R. auricularia *complete their life-cycles in anatid birds, three species require fish-eating birds as definitive hosts, one matures in small mammals, one in amphibians and one in a wide range of birds (final host groups broadly defined in Table 2). Faunal richness of trematodes in *R. auricularia *followed the spatial pattern of overall infection with 11 species at Hengsteysee and seven species at Baldeneysee, whereas only two and one species were found at Kemnader See and Harkotsee, respectively.

Component communities in *R. auricularia *comprised of 1-7 species and were dominated by 1-3 species. Overall, five species (marked with a star in Table 2; four maturing in birds) were considered dominant. It is worth noting that one of these, *Trichobilharzia franki*, a causative agent of cercarial dermatitis (swimmer's itch), was detected at Baldeneysee and Hengsteysee, exhibiting a high prevalence in the latter (Tables 2-3).

### Larval communities in *Lymnaea stagnalis*

The overall prevalence of patent larval trematode infections in *L. stagnalis *was slightly lower than that for *R. auricularia *(15.1%). Snail populations were abundant only in Baldeneysee which is reflected in the larger number of samples available for quantitative comparisons (22-51 snails, see Table 1); sample size and the prevalence of infection were not correlated. Overall prevalence of infection was high at Harkortsee and Kemnader See (21.7 and 18.8%, respectively) and somewhat lower at Baldeneysee and Hengsteysee (14.1 and 9.1%, respectively, Table 2).

*L. stagnalis *in the Ruhr River reservoirs was infected with six trematode species of five families: two species of the family Echinostomatidae and one species each of the families Diplostomidae, Plagiorchiidae, Schistosomatidae and Telorchiidae (Table 2). *L. stagnalis *shared three species with *R. auricularia *(*Echinoparyphium recurvatum*, *O. ranae *and *Plagiorchis elegans*; the former two identified as dominant in trematode communities in *R. auricularia*). Three of the species infecting *L. stagnalis *complete their life-cycles in a wide range of anatids, one species requires fish-eating birds as definitive hosts, one matures in amphibians, and one in a wide range of birds (final host groups broadly defined in Table 2). *Trichobilharzia szidati*, another species known to cause cercarial dermatitis (swimmer's itch), was detected at Baldeneysee although with a moderate prevalence (Table 2). The trematode fauna was relatively species-rich at Baldeneysee (five species) in contrast to the rest of the water bodies studied (one and two species).

Component communities in *L. stagnalis *were species-poor (1-2 species) and were occasionally dominated by a single species, *D. pseudospathaceum *(single community at Harkortsee). This species was the only parasite of *L. stagnalis *found in snail samples from all four reservoirs.

### Between-reservoir patterns of variation in infection

We used the data from the pooled samples by reservoir in order to compare distributional patterns of the larval trematodes among reservoirs for each host species. There were significant differences between reservoirs with respect to the overall prevalence of infection of *R. auricularia *and the prevalence of three of the five dominant species which were present and/or exhibiting higher infection levels at Baldeneysee and Hengsteysee in concordance with the distinctly higher richness recorded at these water bodies (Table 2). Of the dominant species, only *D. spathaceum *had a wider distribution among reservoirs (present in three, dominant in one community at Hengsteysee). There was a significant positive correlation between the overall prevalence of infection and two environmental variables (dissolved oxygen concentration, r_s _= 0.591; and pH, r_s _= 0.423, both p > 0.05; however, the latter was not significant for the reduced dataset).

In contrast, the overall prevalence of infection in *L. stagnalis *did not show significant variation between reservoirs and was not correlated with any of the environmental variables measured. The only dominant species in this host, *D. pseudospathaceum*, was found in all four water bodies and with a significantly higher prevalence and dominating communities sampled at Harkortsee and Kemnader See (Table 2).

The overall probability of infection was strongly dependent on snail size and exhibited a significant association with the levels of dissolved oxygen and pH for both species datasets whereas the temperature at the time of sampling appears to have significantly influenced the collection of infected *R. auricularia *only (Table 3). Reservoir of origin and water conductivity did not exhibit significant association with the probability of infection of both hosts.

**Table 3:** Logistic regression results for the factors significantly affecting the probability of overall infection of *Radix auricularia *(n = 354) and *Lymnaea stagnalis *(n = 171) in the four reservoirs of the Ruhr River.

Snail species	Factor	Estimate	Wald statistics	p
***R. auricularia***	Snail height	0.257	22.92	< 0.0001
	Temperature	0.247	12.59	0.0004
	Dissolved oxygen	0.290	9.03	0.0026
	pH	-0.279	8.01	0.0047

***L. stagnalis***	Snail height	0.188	18.99	< 0.0001
	pH	-1.778	8.33	0.0039
	Dissolved oxygen	0.978	5.83	0.0157

Infection coded 0 (uninfected) and 1 (infected)

### Within-reservoir patterns of variation in infection

Unequal snail density among the studied water bodies affected not only species richness and the distribution of the dominant species as indicated above, but also limited the possibility for assessment of small-scale variations at the component community level, especially for *L. stagnalis*. To achieve this we used data for a total of eight component communities in *R. auricularia *from Baldeneysee (three sites, one sampled twice) and Hengsteysee (four sites) and three communities in *L. stagnalis *from Baldeneysee.

Component communities in *R. auricularia *exhibited a contrasting pattern of variation in the two reservoirs under comparison with low (one species) to moderate species richness (site B3, 3-4 species, see map in Additional file 1) and similar overall infection levels and distributions of the dominant species (with the exception of *O. ranae *found only at the second sampling of site B3, Table 3) at Baldeneysee. On the other hand, although a single species was found at two sites each at Hengsteysee, communities at sites He2 and He3 were both species-rich (7 species, see map in Additional file 1). This richness was associated with substantially higher overall levels of infection and significant differences in the distributions of the dominant species (except *O. ranae *not recorded in this reservoir, Table 4). Communities sampled at Hengsteysee also exhibited a much lower predictability (mean similarity based on species presence/absence of 9.8 *vs *31.5% at Baldeneysee).

The mean similarity between species-poor communities in *L. stagnalis *at Baldeneysee was similar to that for *R. auricularia *from the same reservoir (36.5%). No significant differences were detected in the overall prevalence or the distribution of the dominant species, *D. pseudospathaceum*, which showed moderate prevalences at sites B1 and B3 (see map in Additional file 1 and Table 4).

**Table 4:** Component communities in *R. auricularia *and *L. stagnalis *(reduced dataset) and within-reservoir variations in the levels of larval trematode infections (contrasts in overall prevalence and the prevalence of the dominant species).

Snail species	Trematode species	Prevalence (%)				Significance of differences (p)
	**Baldeneysee**	**Site B1**	**Site B3a**^§^	**Site B3b**^§^	**Site B6**	
		(n= 20)	(n = 76)	(n = 20)	(n = 22)	

***R. auricularia***	Overall prevalence	15.0	22.4	31.6	18.2	ns
	*O. ranae*	-	-	10.5	-	0.018
	*D. spathaceum*	-	-	5.3	-	ns
	*E. recurvatum*	-	17.1	10.5	18.2	ns
	*P. radiatum*	15.0	2.6	-	-	ns
	*N. attenuatus*	**-**	1.3	**-**	**-**	-
	*Echinostoma *sp.	**-**	1.3	**-**	**-**	-
	
***R. auricularia***	**Hengsteysee**	**Site He1**	**Site He2**	**Site He3**	**Site He6a**	
		(n = 35)	(n = 30)	(n = 50)	(n = 51)	
	
	Overall prevalence	2.9	76.7	44.0	2.0	0.0001
	*T. franki*	-	26.7	18.0	-	0.0001
	*E. recurvatum*	-	23.3	-	2.0	0.0001
	*D. spathaceum*	-	13.3	4.0	-	0.007
	*P. radiatum*	-	-	10.0	-	0.008
	*Echinostoma *sp.	2.9	3.3	2.0	-	-
	*P. elegans*	-	3.3	-	-	-
	*A. burti*	-	3.3	-	-	-
	*T. clavata*	-	6.7	-	-	-
	*H. conoideum*	-	-	4.0	-	-
	*N. attenuatus*	-	-	2.0	-	-
	*I. melis*	-	-	4.0	-	-
	
***L. stagnalis***	**Baldeneysee**	**Site B1**	**Site B3a**	**Site B4**		
		(n = 22)	(n = 51)	(n = 37)		
	
	Overall prevalence	4.5	13.7	8.1		ns
	*D. pseudospathaceum*	4.5	5.9	-		ns
	*E. revolutum*	-	7.8	5.4		-
	*P. elegans*	-	-	2.7		-

^§^Same site sampled twice: on 26 September (a) and on 19 October (b).

### Community structure

**Figure 2:**
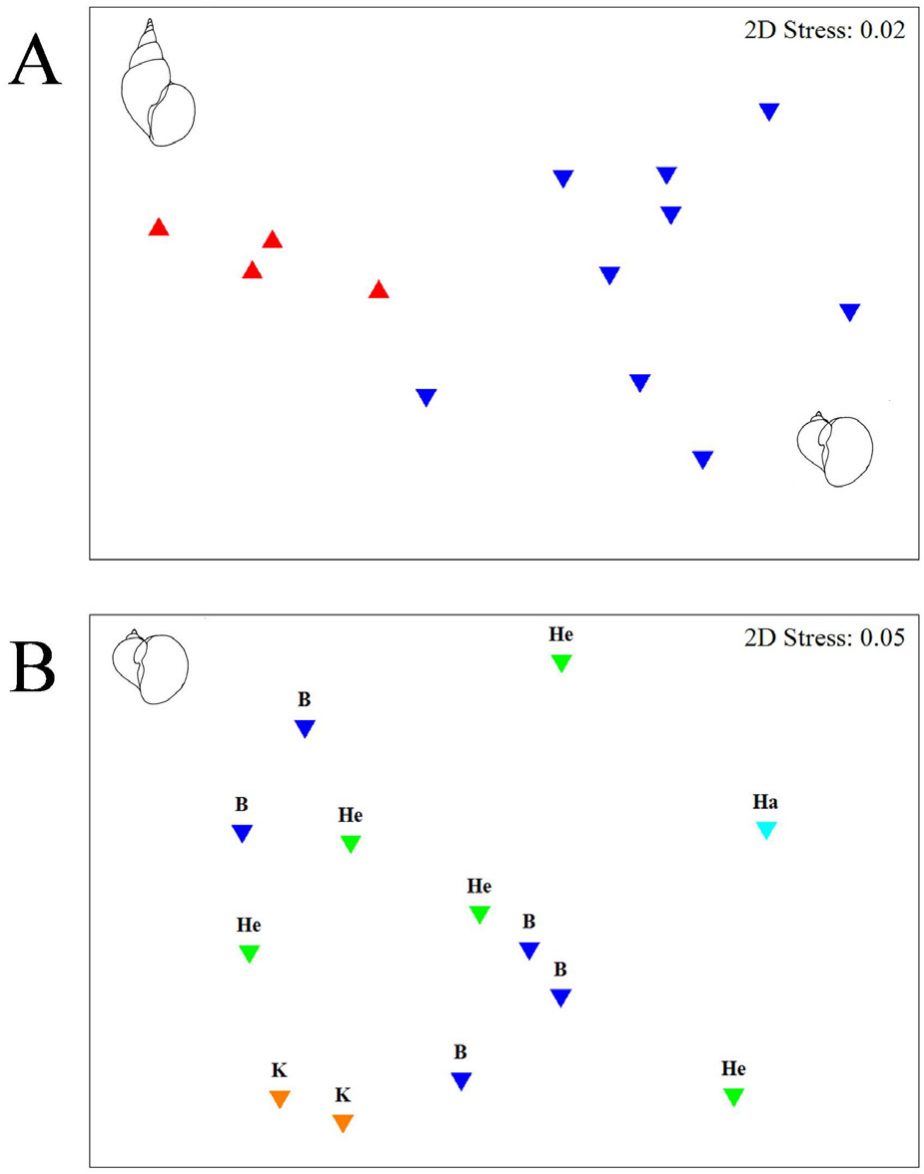
**Two-dimensional MDS ordination plot based on similarity in trematode community structure. A. Reduced dataset for larval communities in *R*. auricularia (blue triangles) and *L*. stagnalis (red triangles). *B*. *R*. auricularia dataset including all samples of more than 10 snails**. Reservoir of origin indicated by different colour. *Abbreviations*: B, Baldeneysee; Ha, Harkortsee; He, Hengsteysee; K, Kemnader See.

The MDS ordination plot of component communities in the two hosts comprising the reduced dataset in Fig. 2A (stress value 0.02) illustrates the significant compartmentalization of community composition by host species (ANOSIM R = 0.522, p = 0.001). There was a higher overall variation of communities in *R. auricularia *which, however, could not be consistently related to the reservoir of origin. In an attempt to increase the representation from the two reservoirs with lower density of *R. auricularia*, Harkortsee and Kemnader See, we included all samples of more than 10 snails. The result of the ANOSIM test although significant for the factor "reservoir" (but with low R = 0.253, p = 0.044), was rather due to the somewhat isolated position of the single community from Harkortsee and the similar composition of the two communities from Kemnader See. On the other hand, communities from the two reservoirs with high density of *R. auricularia *exhibited a wider range of variations as illustrated in the MDS plot (Fig. 2B).

## Discussion

The results of our study demonstrate that the mature reservoir system on the Ruhr River in the west of Germany provides an excellent environment for the development of species-rich and abundant trematode communities in the first intermediate hosts and this was in line with our expectations based on bird abundance. The purely faunistic by-product is nevertheless striking since the total of 15 species of trematodes we detected as a result of a limited sampling of the two mollusc hosts represents half the diversity registered by Faltýnková & Haas [8] in more than 6,000 molluscs belonging to 28 species studied in the southeast of Germany. Furthermore, the faunal richness we observed in the four Ruhr River reservoirs is similar to the 17 species recorded by these authors in the Aischgrund lowland area. Although their data are pooled among the 26 localities sampled and thus not directly comparable to our results based on distinct snail population samples, another remarkable difference is worth noting. Faltýnková & Haas [8] registered a poorer trematode fauna in *R. auricularia *compared with *L. stagnalis *(4 *vs *10 species). Faltýnková [22] and Żbikowska [23] reported a similar relationship (3 *vs *10 spp. and 1 *vs *13 spp., respectively) in small fish ponds near Ceske Budejovice (Czech Republic) and a variety of water bodies in Poland. On the contrary, we observed the reverse faunal richness: 12 *vs *6 species in *R. auricularia *and *L. stagnalis*, respectively. These data tend to support the hypothesis of Wesenberg-Lund [24] that in lakes *R. auricularia *plays a role in the life-cycles of trematodes similar to that of *L. stagnalis *in ponds. In contrast to Wesenberg-Lund's suggestion, Adam & Lewis [25] reported high trematode richness (11 spp.) in a population of *R. auricularia *during the process of its colonisation of a gravel-pit near Wraysbury (UK), whereas the sparse populations of *L. stagnalis *studied in the same area were uninfected. However, the sample sizes of the two snail hosts were not comparable.

Concerning the rates of infection, although data for Asian populations of *R. auricularia *indicate that parasitism may affect large proportions of the snail population (e.g. overall prevalence 18.2% at Lam Tsuen River, Hong Kong [26]), surprisingly little is known for levels of larval parasitism in European populations of this snail host. Adam & Lewis' [25] study depicting prevalence patterns in *R. auricularia *over three years in a single snail population appears so far unique. Notably, their data show high monthly prevalences (typically above 20%) occasionally peaking up to c. 60% (in at least three out of 11 distinct monthly samples, estimated from Figure 1 in Adam & Lewis [25]) in association with the high prevalence of the bird parasite *E. recurvatum *(> 30%). Although overall prevalences observed in communities in *R. auricularia *in the Ruhr reservoirs showed a wider range of variation there were important infection foci, e.g. sites B3, He2 and He3, the latter two showing extremely high infection rates. In contrast, the overall prevalences in *L. stagnalis *(max. 14%) were low compared with those for both *R. auricularia *([25]; this study) and communities of *L. stagnalis *studied in the same season in two small fishponds in the Czech Republic (up to 53 and 65%, Kostadinova, unpublished data). Thus, our data tend to support the "reverse role" hypothesis of Wesenberg-Lund [24] for the two snail hosts in lakes.

There were clear differences in snail population densities associated with reservoir characteristics. Thus the 'large-lake adapted' species *R. auricularia *dominated over *L. stagnalis *especially at Hengsteysee where the macrophyte cover at the sampling sites was poor and all *R. auricularia *were found aggregated on stones. The spread and abundance of *R. auricularia *in the studied reservoir system could also be related to the fact that *Radix *spp. in general can withstand severe water fluctuation [27]. Interestingly, *L. stagnalis *is considered a "calciphile species" not normally found reproducing in waters with less than 20 mg/L calcium [27]. This sensitivity to external calcium concentration can explain the distinctly higher density of this species at Baldeneysee which is characterised by highest mean calcium content of the sediments (10.5 mg/kg *vs *slightly above 4.0 mg/kg at Hengsteysee and less than 4.0 mg/kg in the other two reservoirs [14]) and the higher abundance of the more "softwater" *R. auricularia *at Hengsteysee. Further, although all studied reservoirs show backwater effects as evidenced by the perpetual removal of sediment deposits during the last 20 years, Baldeneysee is characterised by the lowest water flow velocity both in spring and summer (0.07 and 0 m/s, respectively [14]), thus providing a range of lentic habitats suitable for *L. stagnalis*.

Our ability to detect parasites with low prevalence decreases at low population densities since sample sizes also decrease and this is particularly true for mollusc-parasite systems characterised by a remarkable consistency in prevalences (usually within the 5-10% range, see Esch et al. [2]). Although this has inevitably affected our observations, we believe that infection levels may well have reflected real variation among the Ruhr reservoirs and the logistic regression results tend to support this. To the best of our knowledge, this is the first study relating environmental variables with the outcomes of trematode transmission at the level of individual snails in a natural setting. In fact, studies on the impact on the survival and infectivity of parasite transmission stages of environmental variables have rarely been undertaken in laboratory and then only in the case of a few trematodes (reviewed by Pietrock & Marcogliese [28]). In addition to the well-known strong association between infection and snail size (reviewed in [29]), our results indicate that oxygen content may play an important role for trematode survival and/or infectivity especially in areas with poor water column oxygen conditions (e.g. *L. stagnalis *dataset, predominantly sampled at Baldeneysee) and, therefore, be of importance for the distribution of larval trematode infections among reservoirs which differed significantly with respect to this environmental variable. The strong correlation between oxygen content and conductivity explains the exclusion of the latter variable from the models. Further, although hydrogen ion concentration varied within a narrow range, pH was also identified as an important factor with a negative effect on the probability of infection.

We expected a substantial homogenisation of trematode distribution accross reservoirs which would reflect an even distribution of the limited number of bird species at the relatively small spatial scale of the study (Table 1, Additional file 1). However, we observed at Baldeneysee and Hengsteysee, in addition to differential snail densities, the highest trematode richness and this was in sharp contrast with the other two reservoirs. Trematode diversity and prevalence are directly associated with bird diversity and abundance (*e.g. *[30-32]) and both bird species richness and maximum densities increase with nutrient load [33]. Therefore, the diversity and composition of larval trematodes in the bird-parasite dominated (87% of all species and 83% of the dominant species) snail systems studied by us appear to reflect an advanced eutrophication at Baldeneysee and Hengsteysee in line with the predictions for parasites in eutrophic water bodies in the classical study of Wisniewski [34]. In support of our findings, Podraza et al. [14] considered the total phosphorus content of the sediments of Hengsteysee characteristic for eutrophic lakes and recorded more than twice as high levels at Baldeneysee. Furthermore, the two infection foci at Hengsteysee are located in a shallow stagnant bay (sites He2 and He3, see map in Additional file 1) affected predominantly by the inflow from the Lenne River which includes the inflow from a nearby wastewater treatment plant of a paper mill.

However, the two snail-trematode systems depicted different prevalence patterns among reservoirs. The sharp differences in the overall prevalence and the prevalence of three out of five dominant species in *R. auricularia *were in contrast with the more homogeneous overall prevalence between reservoirs in *L. stagnalis*. The pattern observed in the latter host-parasite system is probably associated with the substantially lower overall trematode richness and the higher prevalence of the only dominant species, *D. pseudospathaceum *in Harkortsee and Kemnader See. In spite of the variability observed, the domination of parasites maturing in birds with respect to both richness and prevalence, is a common feature of the two snail-trematode systems studied in the Ruhr reservoirs. Although we have selected a very high threshold prevalence value for dominance status, the number of dominant species appears unprecedented in view of the typically low prevalences of larval trematodes in molluscs [1].

Remarkably, the life-histories of five out of the six dominant species appear to depict two aspects of progressive eutrophication in this mature reservoir system. First, the relatively homogenous prevalences of the two *Diplostomum *spp. completing their life-cycles in gulls and the specific parasite of cormorants *P. radiatum*, indicate increased densities of their most suitable intermediate hosts, cyprinids, which are characteristic for eutrophic conditions. Although no records are available for *P. radiatum*, increased infections of *Diplostomum *spp. in fish were related to progressive eutrophication in the Lake Constance (Germany) [35]. The rates of infection with the three species associated with cyprinids and fish-eating birds in the Ruhr reservoirs studied are within the upper ranges reported so far (typically from pond systems; see [9,21,36,37]).

The high infection rates of the second group of dominant species, *E. recurvatum *and *T. franki*, which mature in anatid birds, suggest parasite proliferation associated with another aspect of lake eutrophication, *i.e. *the dominance of anatids and *Anas platyrhynchos *in particular [34]. High eutrophication of water reservoirs, accompanied by colonisation by snails and nesting ducks, is considered as one of the most important factors for the increase of outbreaks of cercarial dermatitis worldwide [38,39]. Although *Trichobilharzia *spp. infection rates in lymnaeids are typically low, ranging between 0.3 and 5.2% in Europe [9], two of the most diverse sites in our study were also important foci of *T. franki *showing prevalences as high as the maxima recorded (22% and 26% in the Czech Republic [40] and South Germany [41], respectively). It has been suggested that these high infection rates may have resulted from disturbances in the ecological balance of the lakes leading to increased snail densities [39,42] and our data tend to support this. An added factor in our system is the presence of dense mats of *E. nuttallii *on water surface attracting large numbers of plant-grazing anatids and cyprinids and thus facilitating trematode transmission (see bird aggregations outlined in Additional file 1). It is worth noting that cases of cercarial dermatitis have been recorded at Hengsteysee.

One important result of our study is that component community composition and structure provided evidence that larval trematodes in *R. auricularia*, and to a much lesser degree in *L. stagnalis*, may have reflected spatial bird aggregations on the small-scale within-reservoir study especially with respect to the focal occurrence of parasites. Thus, at least three foci exist in Baldeneysee and Hengsteysee that support diverse communities involving up to four different groups of vertebrate final hosts (Table 4). The small-scale patterns of variation of component communities in the two snail hosts also indicated differentiation between host-parasite associations in the two study reservoirs. Thus, infection levels in both snail hosts from Baldeneysee were somewhat homogenous and this was in contrast with the substantial variation in communities in *R. auricularia *at Hengsteysee; unfortunately no representative samples were available for *L. stagnalis *from the latter. One possible explanation is that the larger size of Baldeneysee levels down the environmental instability e.g. water level and temperature variation which is significant at Hengsteysee as it serves as a lower reservoir for a pumped-storage hydropower plant. The bird sanctuary Heisingen at Baldeneysee also supports important numbers of breeding birds. These factors in combination may tend to homogenise trematode communities in molluscs. We are tempted to suggest that the contrasting patterns of variation in communities in *R. auricularia *may be associated with the more advanced eutrophication at Baldeneysee thus offerring support for another insightful prediction of Wisniewski [34]*i.e*. that the distribution of hosts and parasites in an eutrophic water body "leads, as a rule, to the possibility of the hosts being invaded in the same degree at all points at the lake". A wider small-scale sampling at the four Ruhr reservoirs would be influential in testing this prediction.

## Conclusions

In conclusion, we suggest that trematode communities in the lake-adapted *R. auricularia *are better suited for monitoring the effect of environmental change on host-parasite associations in the mature reservoir system on the Ruhr River and other similar systems due to the high density of this host and its important role in trematode transmission in lakes. Whereas variations in trematode community diversity and abundance may indicate the degree of eutrophication on a larger scale (among reservoirs), the infection rates of the two life history groups of dominant species, the 'cyprinid' and 'anatid' assemblages, may be particularly useful in depicting environmental variability, eutrophication effects and infection 'hot spots' on smaller spatial scales.

## Competing interests

The authors declare that they have no competing interests.

## Authors' contributions

APO, MS and BS conceived the study and designed the sampling strategy. MS, CS and APO carried out the sampling, parasite screening and identification, and drafted the MS. AK and APO carried out the main statistical analyses. BS, APO and AK coordinated the study and revised the final draft of the manuscript. All authors read and approved the final manuscript.

## Supplementary Material

Additional file 1 **Maps of the four reservoirs on the Ruhr River with indication of the sampling sites, bird aggregations and photos**. Click here for file
