# Supplementary material for: Larval trematode communities in Radix auricularia and Lymnaea stagnalis in a reservoir system of the Ruhr River
Source: Parasit Vectors. 2010 Jun 24;3:56. doi: 10.1186/1756-3305-3-56 (PMC2910012; doi:10.1186/1756-3305-3-56)
Supplement: Additional file 1 — Maps of the four reservoirs on the Ruhr River with indication of the sampling sites, bird aggregations and photos. [file 1756-3305-3-56-S1.DOC]

## Additional file 1

**Baldeneysee**

**Sites B1, B2, B3 and B6**


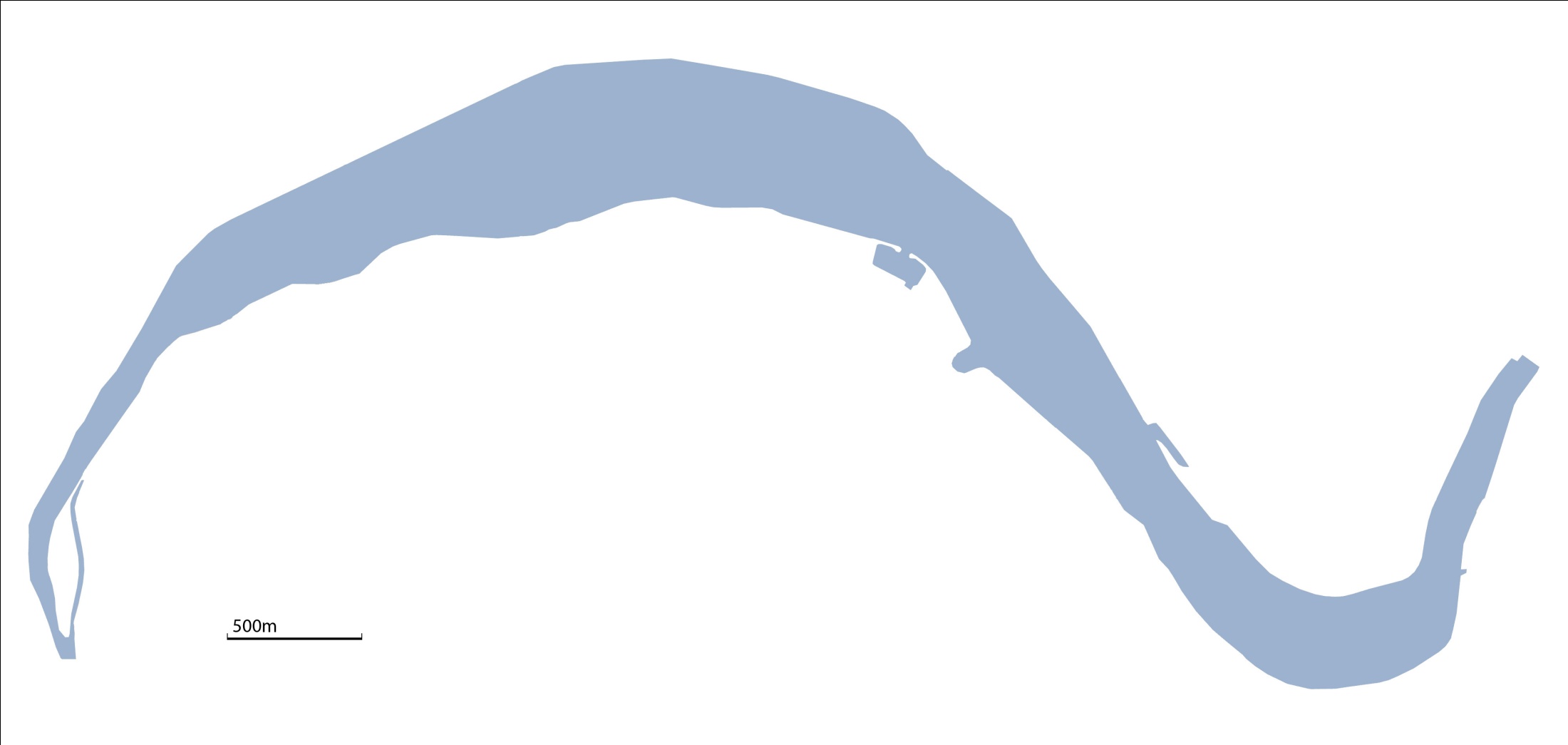


Phalacrocorax carbo (6)

*Fulica atra* (>60)

*Cygnus olor* (2)

*Anas platyrhynchos* (<15)

*Anas platyrhynchos* (c. 20)

*Tachybaptus ruficollis* (1)

B1

B2

B3

B6

**Sites B5 and B7**


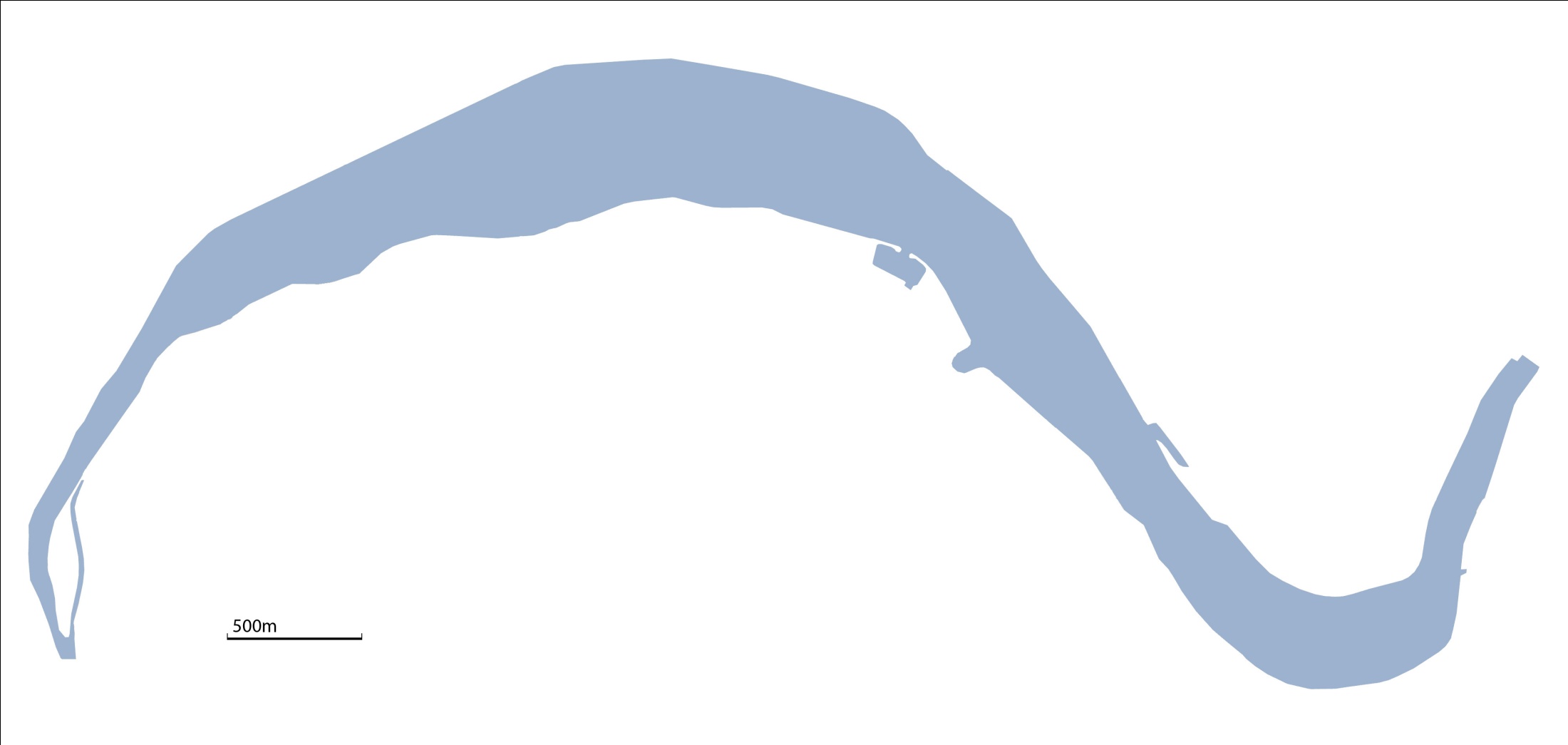


*Anas platyrhynchos* (>50)

*Larus ridibundus* (c. 10)

*Cygnus olor* (6, on jetty)

B5

B7

**Site B4**
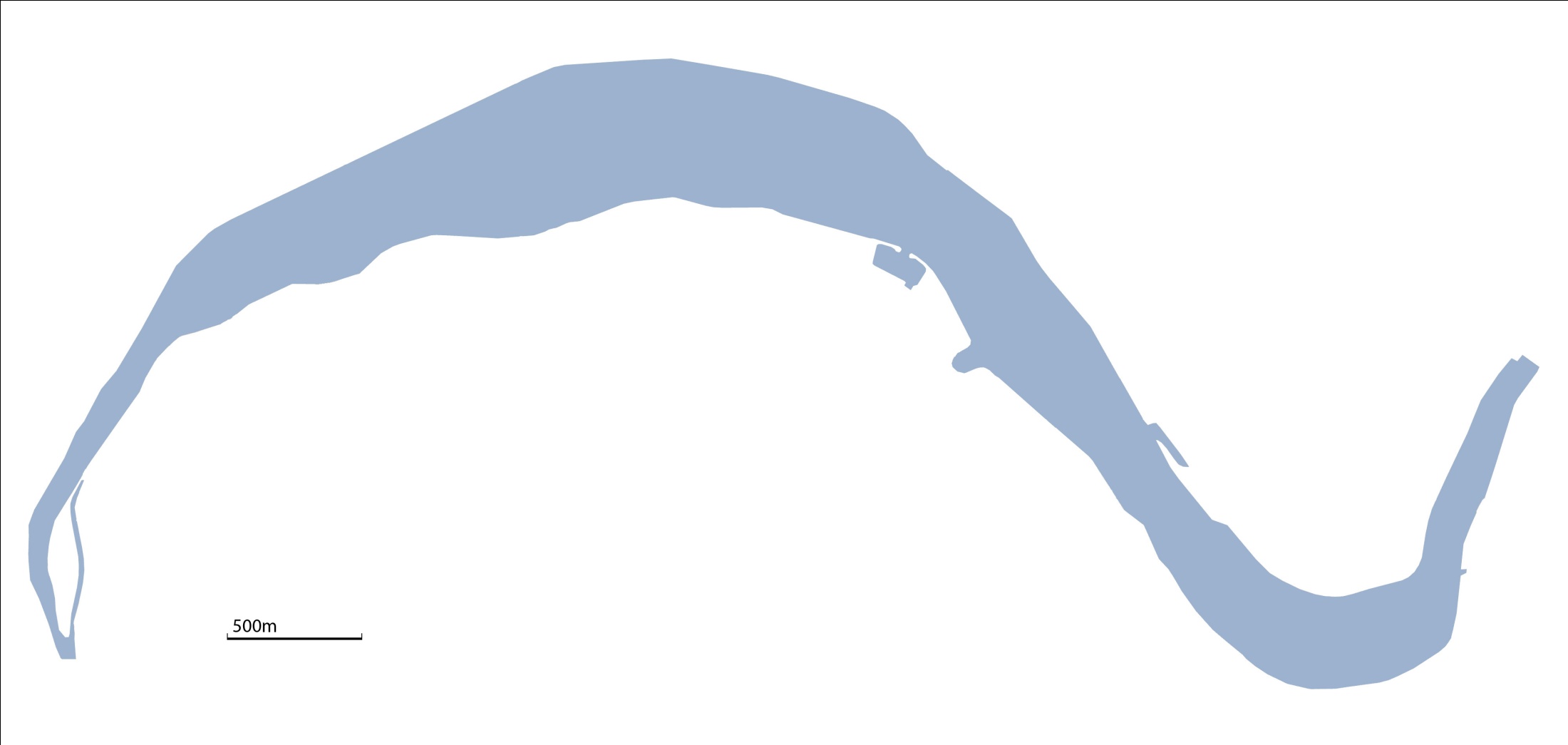


*Cygnus olor* (c. 30, 100 - 200 m off shore)

*Fulica atra* (80-100)

B4

**Kemnader See**

**Site K1**


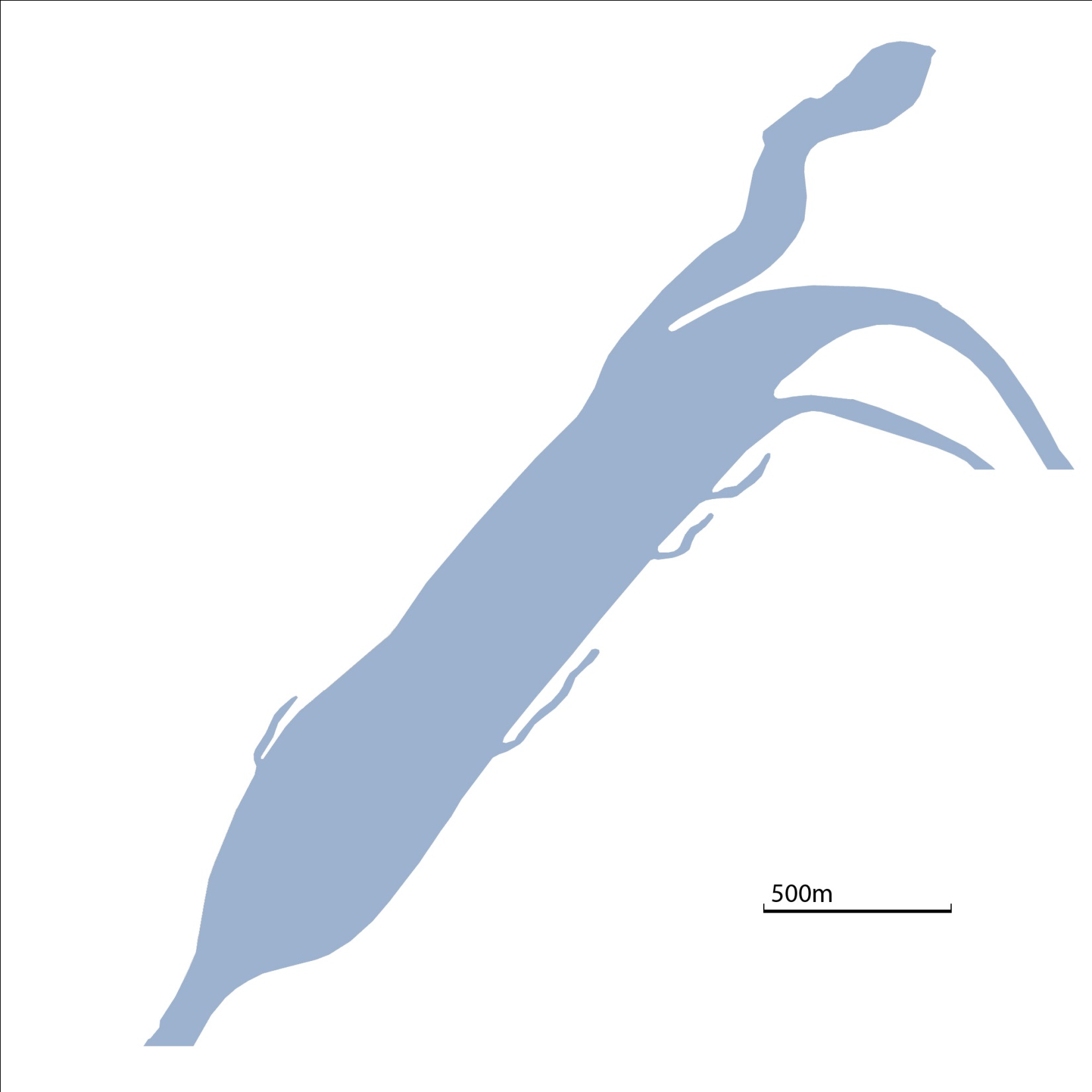


*Anas platyrhynchos* *Alopochen aegyptiacus*

*Fulica atra*

*Cygnus olor* (3)

*Fulica atra* (>50, on the reservoir)

K1


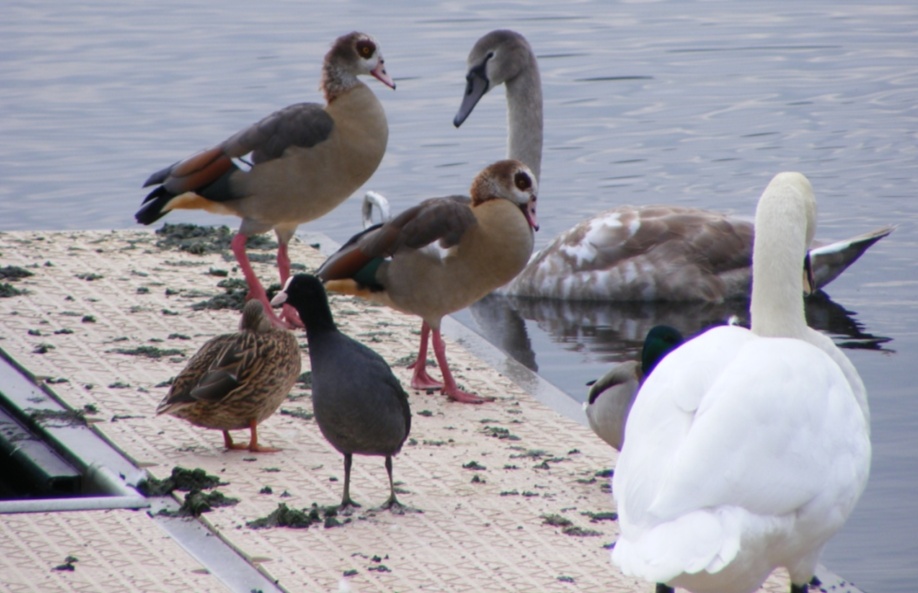


*Anas platyrhynchos*, *Alopochen aegyptiacus*, *Cygnus olor* and *Fulica atra* on a jetty close to K1

**Site K3**


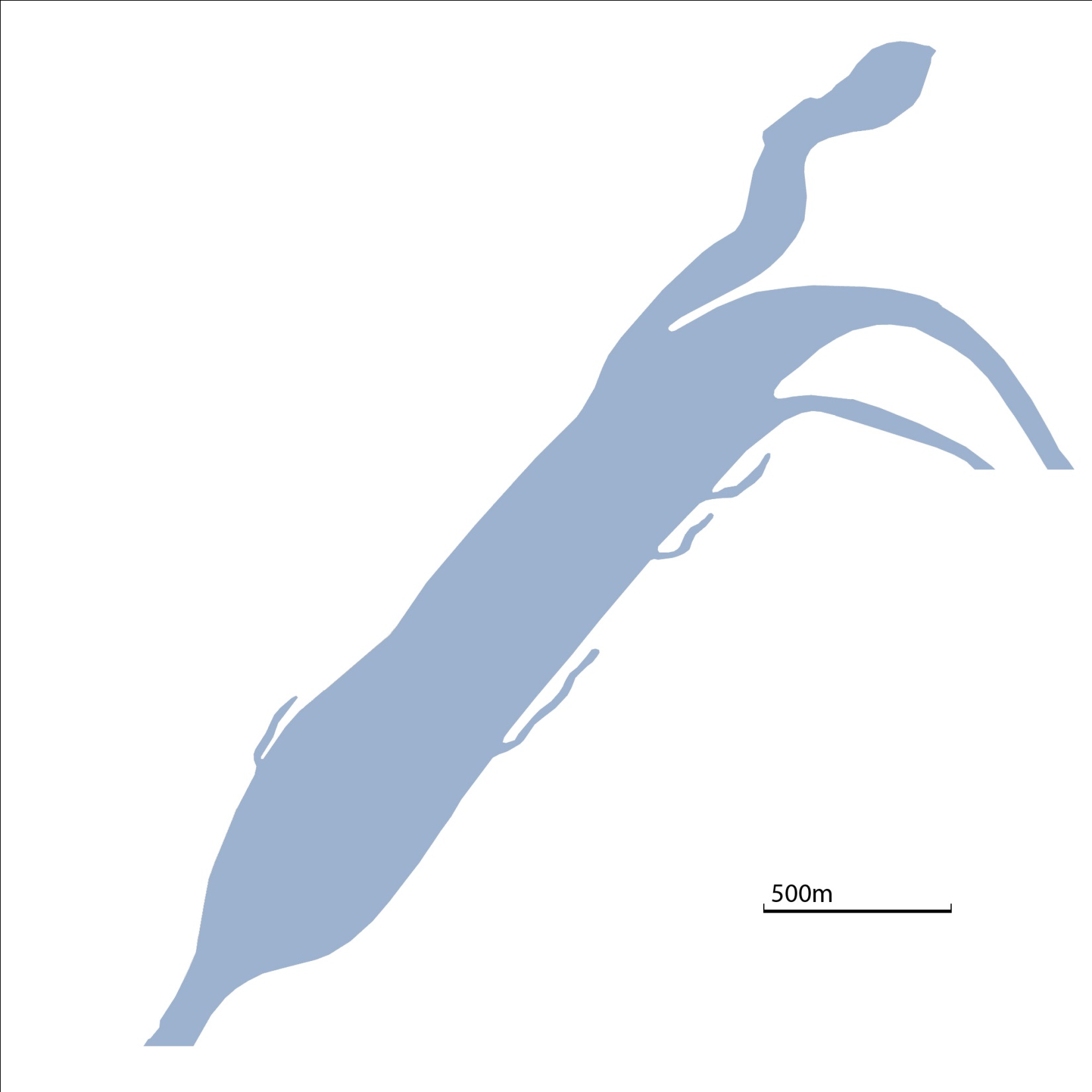


No birds close to site K3

*Branta canadensis* (c. 20)

*Anas platyrhynchos*

*Larus ridibundus* (>40)

*Ardea cinerea* (1)

*Cygnus olor*

*Branta canadensis* (2)

*Podiceps cristatus* (2)

K3


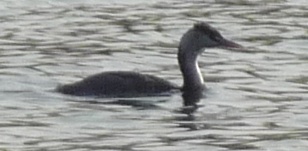

*Podiceps cristatus* close to K3


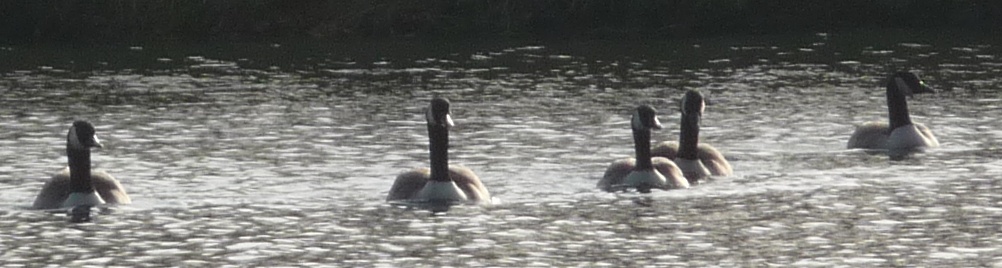

*Branta canadensis* close to site K3


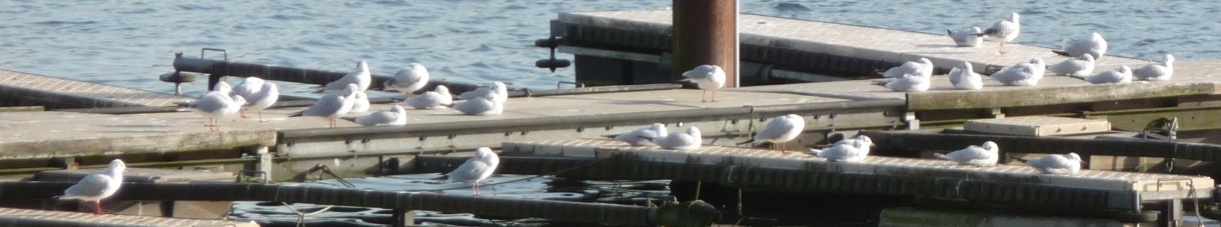

*Larus ridibundus* on a jetty close to site K3


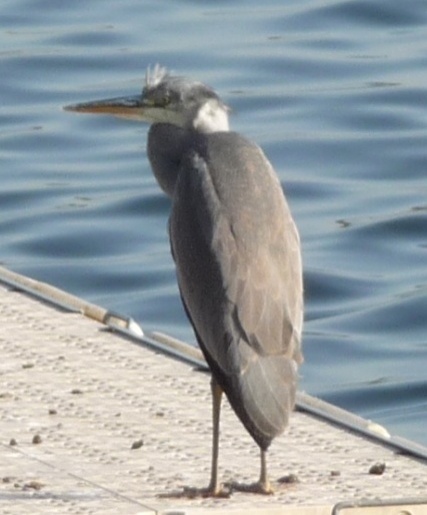

*Ardea cinerea* on jetty close to site K3

**Harkortsee and Hengsteysee**

**Sites Ha1 and Ha2**


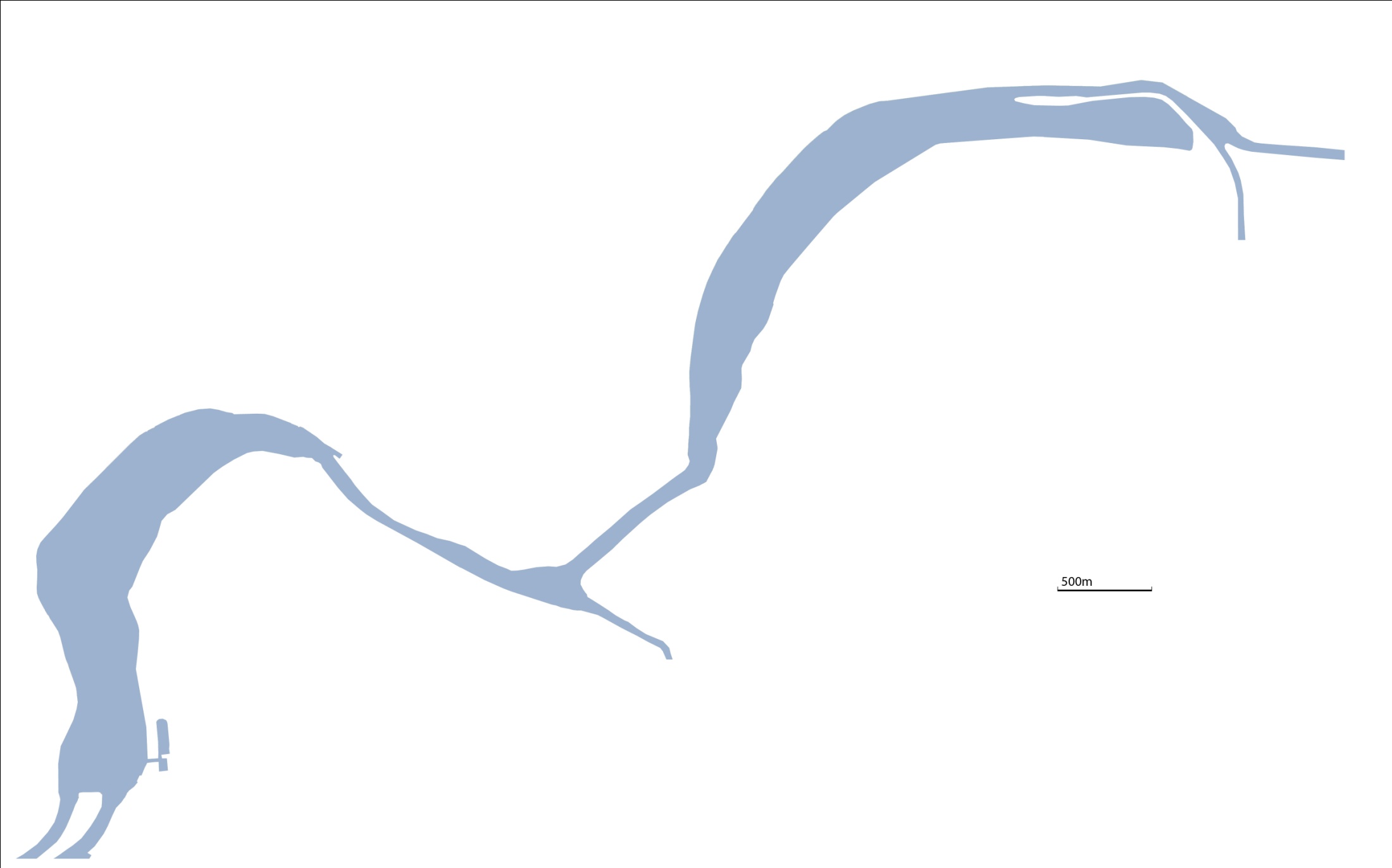


*Fulica atra* (>100, close to shore)

*Anas platyrhynchos*

*Larus ridibundus*

*Cygnus olor* (2)

*Podiceps cristatus*

No birds close to sampling sites Ha1 and Ha2 at time of survey

*Branta canadensis* and Phalacrocorax carbo common in this area according to residents

Ha1

Ha2


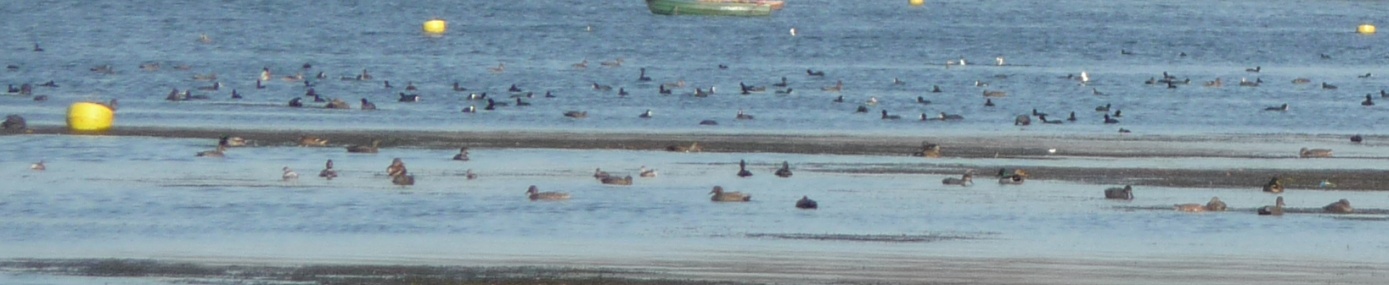


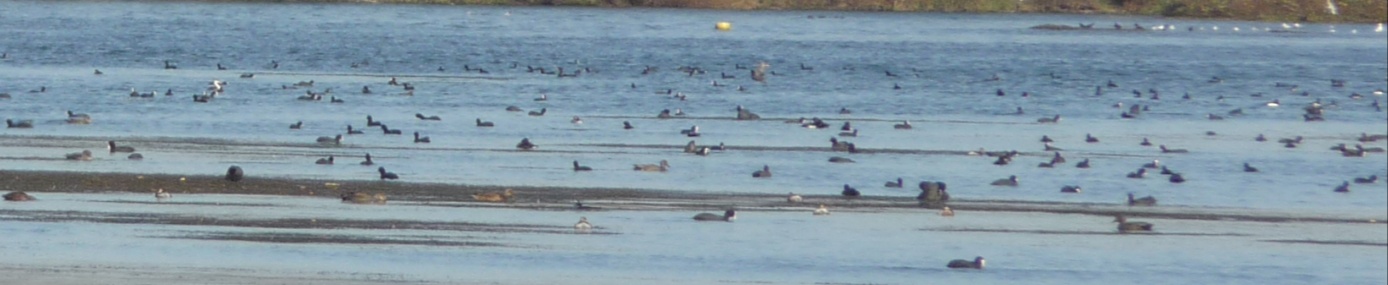
 *Anas platyrhynchos* and *Fulica atra* on Harkortsee

**Sites He1, He4 and He6**


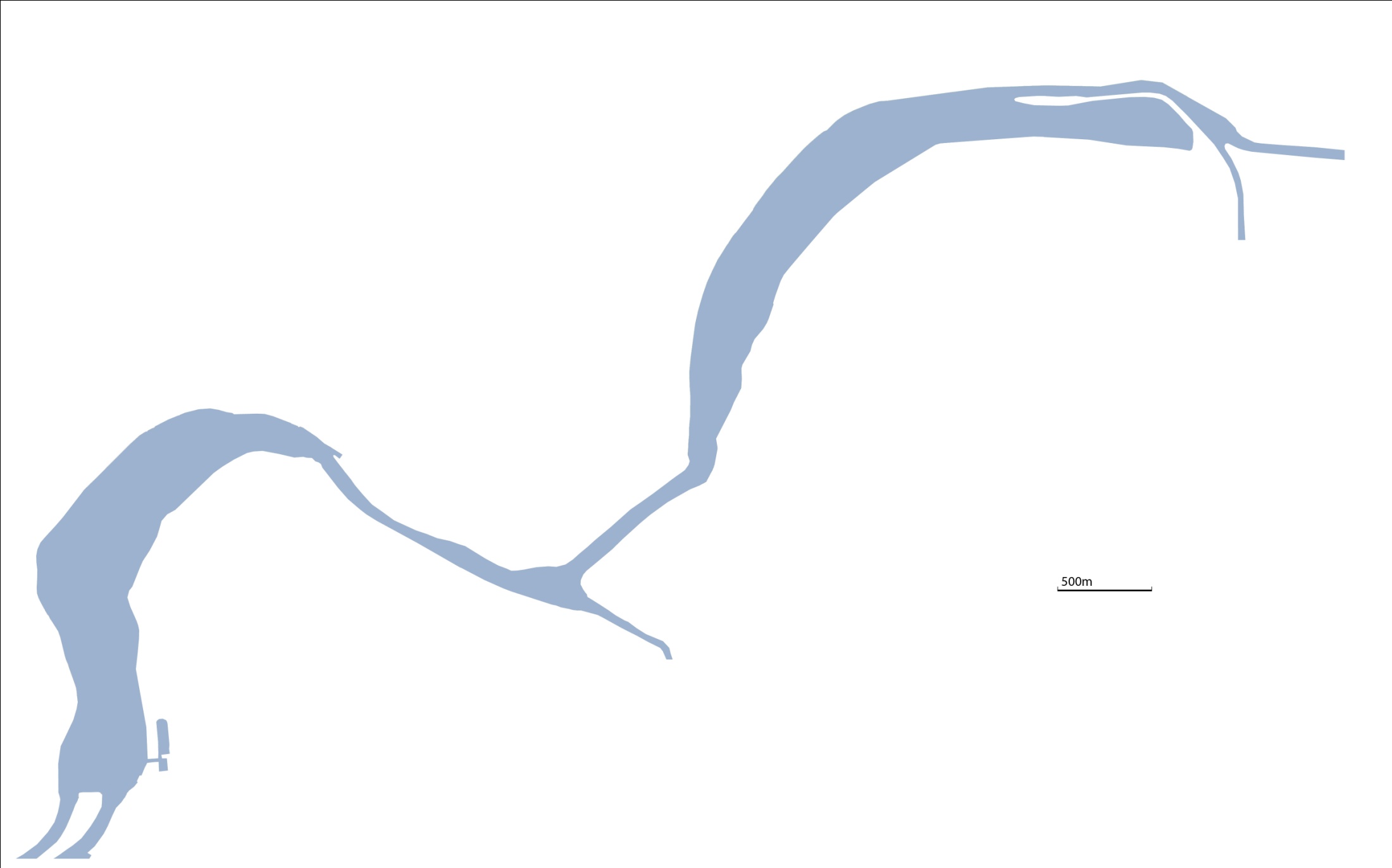


Phalacrocorax carbo (5)

*Cygnus olor* (ca. 40)

*Fulica atra* (>100)

*Larus ridibundus*

*Anas platyrhynchos* (close to shore)

No birds at time of survey;

Water level fluctuation due to power plant (see below)

He6

He4

He1


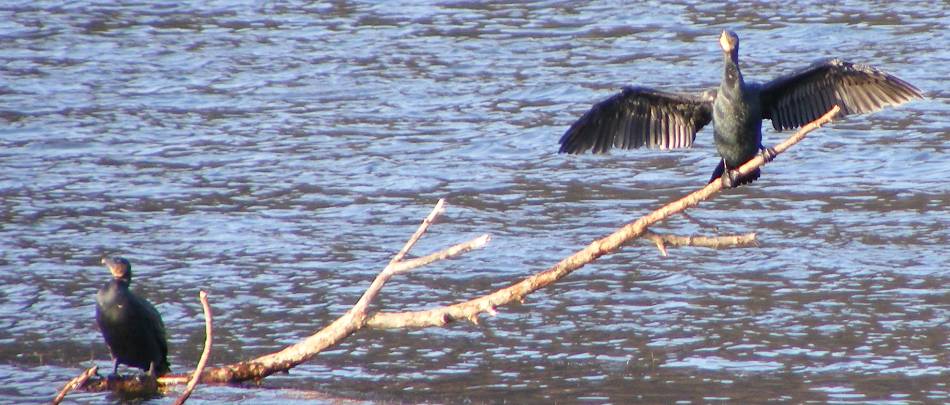

Phalacrocorax carbo on Hengsteysee (between sites He1 and He4)


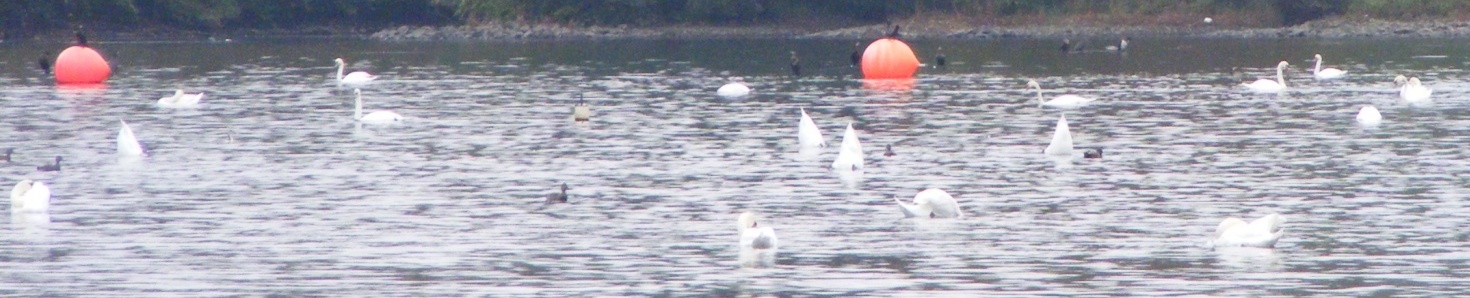
 *Cygnus olor*, Phalacrocorax carbo, Fulica atra and ducks close to site He1


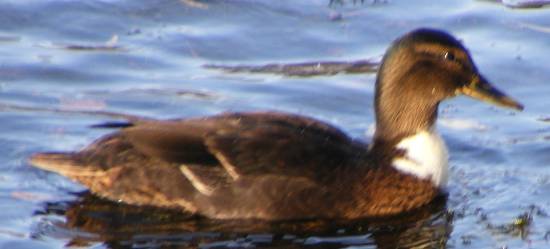

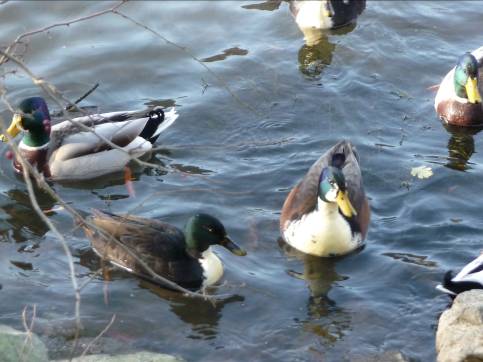

Domestic duck close to site He4 *Anas platyrhynchos* and domestic ducks close to site He4

**Sites He2, He3 and He5**


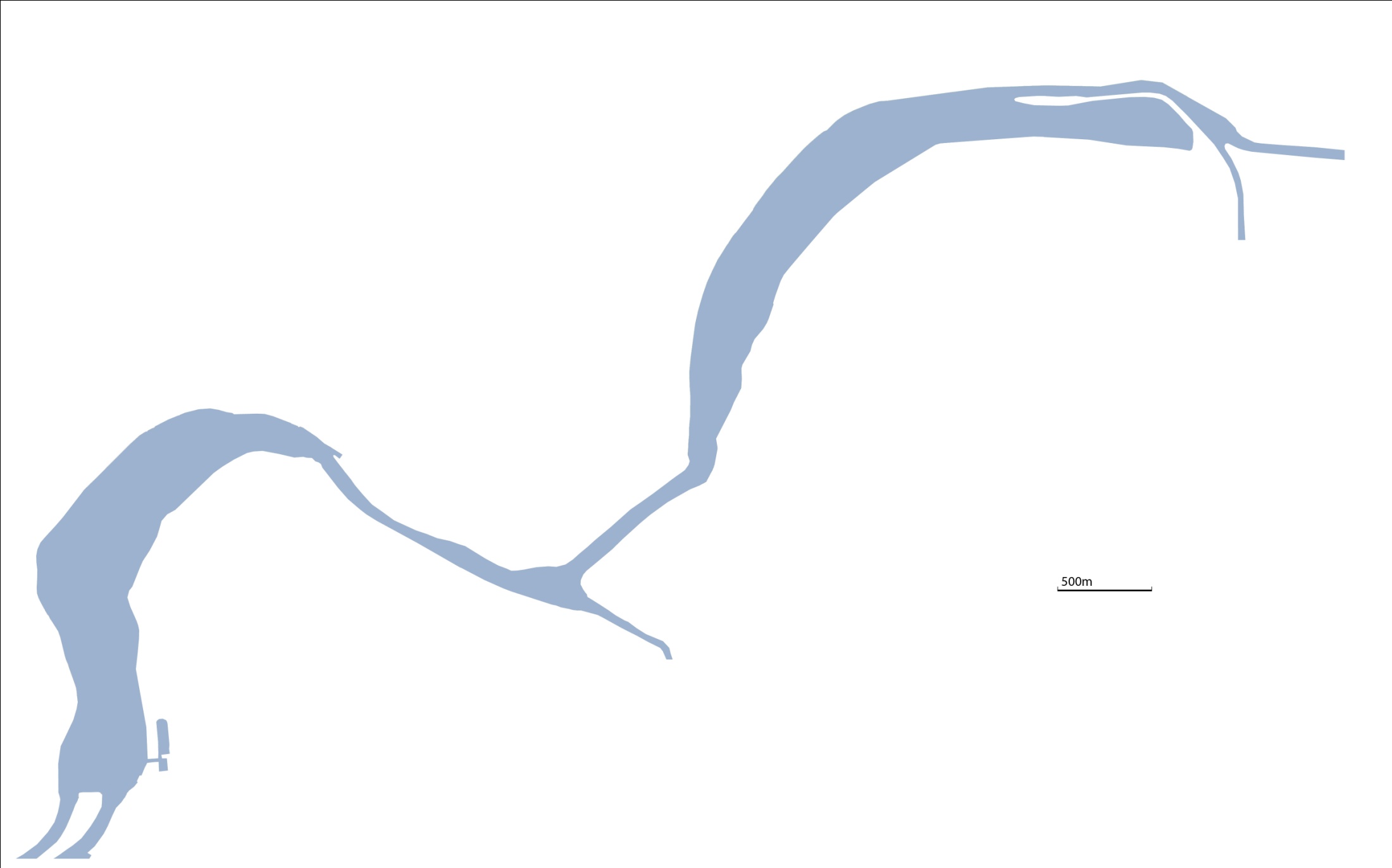


No birds at time of

survey;

Water level fluctuation

due to power plant

(see below)

*Cygnus olor*

He5

He3

He2


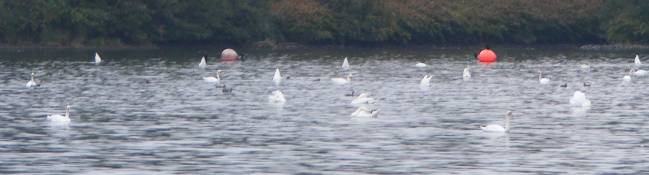

*Cygnus olor*, *Fulica atra* and *Phalacrocorax carbo* close to sites He2 and He3


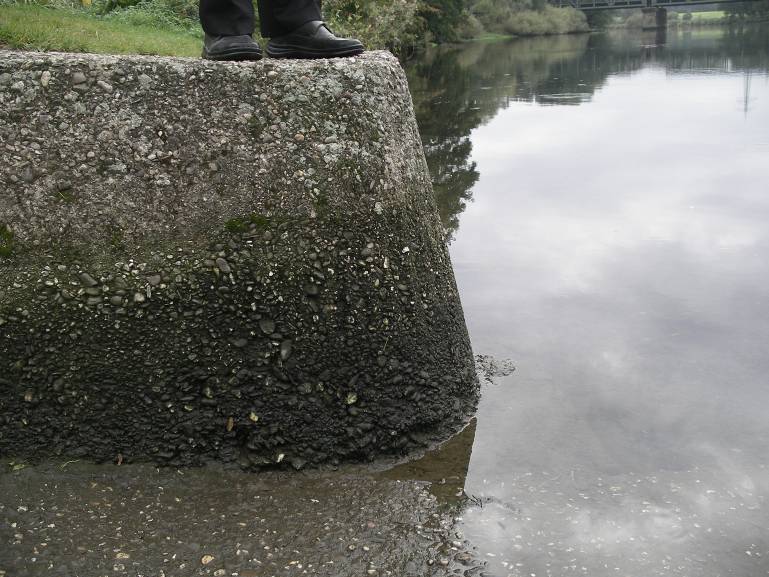

Site He5

Water level fluctuation

due to operation of the pumped-storage hydropower plant at Hengsteysee
